# Financing conditions of renewable energy projects – results from an EU wide survey

**DOI:** 10.12688/openreseurope.13969.1

**Published:** 2021-11-12

**Authors:** Robert Brückmann, Agustin Roth, Moïra Jimeno, Jörn Banasiak, Mak Đukan, Lena Kitzing, Vasilios Anatolitis

**Affiliations:** 1 Policy Consulting, eclareon GmbH, Berlin, 10117, Germany; 2 DTU Wind Energy, Technical University of Denmark, Lyngby, 2800 Kgs., Denmark; 3 DTU Management, Technical University of Denmark, Lyngby, 2800, Denmark; 4 Fraunhofer Institute for Systems and Innovation Research ISI, Karlsruhe, 76139, Germany

**Keywords:** Renewable Energy Auctions; WACC; Costs of Capital; Renewable Energy Financing; Cost of Debt; Cost of Equity; Renewable Energy Support

## Abstract

This data note aims to present a dataset with values for financing conditions for renewable energy projects in Europe. This includes weighted average cost of capital, cost of debt, cost of equity, debt share, debt service coverage ratio and loan tenors. The dataset was elaborated in the framework of the "Auctions for Renewable Energy Support II" project (AURES II). The main goal of the AURES II project is to provide policy support to the Member States of the European Union in order to improve the effectiveness and cost-efficiency of auctions for renewable energy support. As part of the AURES II project, an extensive survey (structured interviews) was conducted between September 2019 and April 2020 with different stakeholders involved in the renewable energy industry, such as banks, project developing companies, and investment funds, among others. The technologies covered were solar photovoltaics (PV), wind onshore, and wind offshore. Interviewees were asked to provide values for financing conditions for specific projects (for certain cases, country estimates or ranges of values were provided). Spain, Portugal, Greece, Germany, and Denmark were selected as focus countries, for which the interviews also included qualitative questions to discuss the observed quantitative data in these countries. The presented data has been used as the main input to elaborate an AURES II report on renewable energy financing conditions in Europe.

## Plain language summary

In this document we present quantitative data related to financial variables of renewable energy projects (wind and solar power), i.e., variables that show under which conditions investors and companies get funds to build and operate their projects.

The data was collected through multiple interviews conducted between September 2019 and April 2020 with different actors involved in renewable energy projects, for example, bankers or investment funds, among others. We asked them about specific values of key financial variables, such as the cost of capital, which represents an average of the total financing costs that an investor has when developing a renewable energy project. For example, when building a wind farm, a lot of capital is needed in the initial stages, hence, the project developer needs to obtain funds from banks, apart from using own capital. The interviews helped us understand how high these values are and how they have decreased over time for specific technologies and countries in Europe.

## Introduction

This data note aims to present information related to the collection of data for one of the work packages (WP) of the Auctions for Renewable Energy Support II project (AURES II). AURES II is a European research project on auction design for renewable energy (RE) support in the Member States of the European Union (EU). The main goal of the AURES II project is to provide support to policymakers in the EU in order to improve the effectiveness and cost-efficiency of auctions for RE support. Further information can be found on the official project website: www.aures2project.eu. 

In particular, WP5, which deals with financing conditions and RE auctions, consists of three main tasks. First, the consortium conducted research to identify the auction design elements that have an impact on RE financing conditions. Second, building on this first set of findings, in task two, an extensive survey (structured interviews) regarding cost of capital data of RE projects in the EU was conducted. Third, the project delivered auction design recommendations that could lead to more favorable financing conditions.

The scope of the data note is focused on task two of WP5, i.e., the cost of capital data collection ( Brückmann *et al.*, 2021). As part of the deliverables of the AURES II project, a report was written to present the main results related to trends and main insights on cost of capital (also known as weighted average cost of capital - WACC); the significance of certain explanatory variables; and cash-flow impacts on support cost in auction and non-auction environments ( Roth *et al.*, 2021).

## Methods

### Collecting financing data across the EU

The cost of capital data was collected through structured interviews with stakeholders in EU Member States. The research team conducted the majority of the interviews in the local language of the respective country, or in English as default. The team consisted of Master and PhD students across the European Union, which cover all the official EU languages, and consisted of 18 members. A five-step methodology was followed in collecting the data, as explained below.

***Defining the scope.*** First, a distinction was made between focus and non-focus countries. When conducting the interviews, the research team used two different survey templates. On the one hand, for non-focus countries, the template was limited to obtain quantitative data on specific financial variables for renewable energy projects. On the other hand, the template for focus countries was designed to allow for more in-depth research. Hence, it included not only the quantitative questions on financial variables but also other qualitative questions to trigger discussions about the evolution of the costs of capital and the impact of auctions. Both templates are available in the extended data ( Brückmann *et al.*, 2021).

Five EU Member States were selected as focus countries: Denmark, Germany, Greece, Portugal, and Spain. When selecting the focus countries, an energy policy exclusion criterion was taken into account: only countries that had conducted at least one renewable energy auction were considered. The reason behind this criterion is that one of the objectives of the AURES II project is to understand the interrelations and inter-dependencies between auctions and financial variables.

At the moment of selecting the focus countries, the following Member States had already conducted auctions: Croatia, Denmark, Estonia, Finland, France, Germany, Greece, Hungary, Ireland, Italy, Lithuania, Luxembourg, Malta, Poland, Portugal, Slovenia, Spain, the Netherlands, and the United Kingdom. From these countries, the research team selected the focus countries considering a geographical balance.

***Identifying the interviewees.*** Second, based on desktop research and experience from previous similar projects, different relevant stakeholders were identified and categorized: banks, project developers, financial experts, utilities, investment funds and others. Although the list of stakeholders involved in the energy transition is much broader, the scope of WP5 was limited to collect project-based quantitative and qualitative cost of capital data, which ultimately defined the categories of actors. The main criteria in including a stakeholder in the research was direct experience with actual projects and their financing - nevertheless, we did not apply any formal criteria, for instance a minimum number of years of work experience. The main reason behind this is practical - interviewee acquisition is challenging, especially if the topic is sensitive, like in our case. Auctions are based on price competition, where costs of capital are key information in final bid formation. Therefore, revealing this to the competition could cause direct financial damage, for instance in the form of sunk costs because of losing a bid. We agreed on the above defined categories based on project-internal review rounds during the development of the survey. Therefore, these categories were verified by other consortium members.

We mapped the stakeholder categories along the project development timeline. During project development, professional project developers conduct the initial economic and technical studies and lead the project through the permitting process. In some cases, larger utilities or energy companies conduct this activity in-house. To decrease technical risks during construction and operation, insurance companies provide projects with insurance packages. These sometimes also hedge projects against price risks, especially if the projects’ remuneration scheme and support contract expose the project to volatile electricity markets. After the project wins support in an auction, bankers finance the projects, typically in project financing deals. When the project is constructed and operational, larger institutional investors and/or private equity funds buy shares in the project - for instance, a pension fund seeking to "green" its investment portfolio. These investors may also finance projects during their development stage. When a stakeholder did not fall within any of these categories, we mapped him or her under the category "other". For example, we interviewed many consultants that provide financial advice but do not fall within any of the above listed categories.

To select the interviewees, the research team conducted extensive desktop research to reach possible interview partners including different kind of stakeholders involved in the financing of wind and photovoltaics (PV) projects, e.g., investors, developers, banks as well as renewable energy associations. This was made by identifying two groups of countries. Countries with wind and PV auctions between 2018 and 2019 (all EU and UK, except for Austria, Belgium, Bulgaria Estonia, Ireland, Croatia, Latvia, Romania, Sweden, Slovakia, Czech Republic and Cyprus) and countries without auctions at that time. In countries where PV and wind auctions have been conducted, possible interview candidates were identified by screening the publicly available lists of auction participants, as well as their renewable energy associations. In these countries, the renewable energy association helped to identify further stakeholders involved in finance of PV and wind projects. In countries where auctions had not taken place yet, the renewable energy associations were contacted to obtain data, as well as to get support to identify potential interviewees involved in finance of wind and PV. Then, the contact information was extracted from the potential interviewees’ websites, including e-mail and phone (in case this information was already available on the organisations’ websites).

Previously, some consortium members were involved in two EU-funded projects (DiaCore ^1^ and Re-Frame ^2^), for which similar data was collected. The professional networks built in these two projects were also used to arrange interviews for WP5 of the AURES II project.

***Designing the survey template.*** Third, the consortium designed and elaborated the questionnaires to be used in the interviews, both for focus and non-focus countries, which can be found in the extended data ( Brückmann *et al.*, 2021). To ensure that the survey asks relevant questions and is structured in a comprehensive and logical manner, we conducted five initial exploratory interviews and adjusted the survey based on the received feedback (the original version of the questionnaire used for the exploratory interviews can be found in the AURES II exploratory interviews template Brückmann *et al.* (2021)).

The questionnaires start first with information on work background and experience of the interviewee, followed by questions on quantitative values for financing variables of RE projects: WACC, cost of debt (CoD), cost of equity (CoE), debt service coverage ratio (DSCR), loan tenor, technology, size of the project, type of financing and time of completed construction for solar PV, wind onshore and wind offshore. The questionnaires asked for values of specific projects. However, if the interviewees did not want to reveal data that could lead to the identification of a concrete project, they were asked to provide general country estimates instead (i.e., an average WACC value for instance, which can be an average estimate of RE projects in the country and in the time considered).

In the five focus countries, we complemented the quantitative section with a semi-structured discussion on the impacts of auctions on the financing variables, the reason behind the changes in their values, and how specific auction design elements affect project financing (see the focus country questionnaire in the extended data ( Brückmann *et al.*, 2021)). First, the interviewees were asked if the cost of debt, cost of equity, DSCR, loan tenor, and debt to equity ratio changed after the introduction of auctions and why these changes occurred. Second, they were asked to provide the main three reasons of the changes and to also rank the reason(s) according to their importance between 0 and 4, where 0 means not important at all (i.e., not applicable), 1 slightly important, 2 important, 3 fairly important and 4 very important. To stimulate a discussion, they were shown an illustration (see focus country questionnaire in Brückmann *et al.* (2021)) that depicts different auction design elements (such as auction volumes, bid bonds, and penalties), and connected them to different stages in the lifetime of a renewable energy power plant. For instance, auction volumes and frequency may affect planning risk during the pre-development and development phases. Material and financial pre-qualification requirements may affect the bidders’ allocation risk (the risk of obtaining support or winning an auction) - stricter requirements increase potential sunk costs. Penalties and realization deadlines could affect the risk of being penalized, while remuneration scheme design affect directly the project revenues and may impact credit risk - namely the debt providers risk of recovering the loan.

***Conducting the interviews.*** Fourth, between November 2019 and April 2020, the consortium conducted interviews in EU Member States (and the United Kingdom), consisting of one interview session per each interviewee. All interviews were conducted via online conference tools or through a telephone call. The average duration of the focus countries’ interviews was around 45 minutes, whereas the average duration for the non-focus countries was around 25 minutes. The templates used for both focus and non-focus countries can be found in the extended data ( Brückmann *et al.*, 2021).

The research team conducted the interviews individually and in groups of two researchers, where this was possible. Due to the large scope of the survey involving all EU Member States, we did not manage to ensure the consistent participation of at least two interviewers. We acknowledge that this is a limitation, as those interviewers that conducted interviews individually might have misinterpreted some answers. In the majority of cases, interviews were not recorded due to confidentiality reasons and the interviewer took personal notes during the course of the interview. However, in few cases, the interviews were recorded (with the prior consent of the interviewees) and the recordings were used to re-listen to the discussion and to note down the main points. In this way, the interviewer had more capacity to engage in the discussion without taking notes. The recording files were automatically deleted afterwards. The overall notes were then used to explain the quantitative survey findings - published in a project report ( Roth *et al.*, 2021).

Arranging interviews and accessing financial variables was challenging because these data are key for private actors and may be considered as a trade secret. Hence, in order to mitigate this challenge, the interviews were conducted under the Chatham House Rule, meaning that the interviews were anonymous and the interviewees’ answers cannot be used to identify them. The details of the participants were never published or disclosed to third parties (inside the AURES II consortium as well as external parties).

The approach used to arrange and conduct the interviews was based on different steps. First, when the researchers contacted the potential interviewees for the first time, they were asked for their consent to participate in an interview concerning the aforementioned financial variables, highlighting that the interview would be anonymous. In the e-mails with the interview invitations, a .pdf document was attached explicitly mentioning that the interview would be anonymized, and that “your answers will never be linked to your identity or your organization” and also that “you can withdraw during or anytime after the completion of the interview before the publication of the study”. Besides, a link to the official website of the Chatham House Rule was included in this document.

During the interview we explicitly noted that the consortium will use all information/data for the purposes of the AURES II project but keeping their identity confidential. We decided not to collect the written consent forms since in most cases interviewees were not willing to sign and send them back. Nevertheless, participants were asked orally before the start of the interview whether they agree to those.

In addition, during the interview, it was again stated that interviewees can withdraw during or any time after the completion of the interview and before the publication of the results.

Ethical approval was not required for this study. Original interviews containing all the data, i.e., the completed questionnaires, have been internally stored on eclareon’s server to prevent access from third parties. Only anonymized versions of interviews were uploaded to the AURES II consortium platform (which is only accessible to AURES II consortium members). Due to confidentiality concerns, we do not include any transcripts and notes in the extended data ^3^.

***Data collection and processing.*** Fifth, all collected data was centrally extrapolated and compiled into an Excel file. To process the data, we used Excel 2016 in these steps.

First, we developed an input template, where each column represents a single question or data value (for instance WACC values), while each row represents one response. To anonymize the data, the responses were given codes - for instance in Austria we collected 11 responses and named them from AT01 to AT11.

Second, instead of providing a specific value for the financing variables, some interviewees gave us a range of estimates, as they were responding to the survey from memory and not by reading a specific value from a financing term sheet. Consequently, we created subcategories that differentiate between the best and worst input per each financing variable and per each survey input. We assigned these sub-values the codes such as "AT01 - best fin" and "AT01 - worst fin". To maintain consistency and structure across the dataset, we did this for all the inputs - regardless if they are a single estimate or a range estimate.

In a third step, we cleaned the input data so that it does not contain space values and that the inputs are inserted using the same number format and/or words (for instance "onshore wind" instead of "wind onshore" or just "onshore").

Fourth, we summarized each financing variable into minimum, maximum and average data input per country and technology. We did not calculate median and upper/lower quartile values and outliers since the dataset contains a limited number of inputs per country and technology.

Finally, we excluded from the available dataset the country-technology cases for which we have collected less than 3 survey inputs. We do this to maintain data confidentiality, as the low number of data inputs could enable the users of the Data Note to identify a survey respondent. This led to the exclusion of the following country-technology cases: wind onshore in Bulgaria, Cyprus, Hungary, Ireland, Slovakia, Slovenia, and the United Kingdom; solar PV in Bulgaria and Cyprus; and wind offshore in Ireland, Latvia, and the Netherlands.

Although we have collected 240 survey inputs, we show in the available dataset ( Brückmann *et al.*, 2021) the information for only 206 estimates, because of this exclusion criterion ^4^. While this decreases the number of countries the Data Note covers, it increases the robustness of the data and we can ensure the confidentiality of the interviewees.

### Description of the structure of the dataset

The dataset in ( Brückmann *et al.*, 2021) consists of average, minimum, and maximum values of the survey inputs, resulting in costs of capital and financing conditions data for wind onshore (19 countries), solar PV (10 countries), and wind offshore (four countries):

- Cost of debt (CoD) [min, average, and max]
- Cost of equity (CoE) [min, average, and max]
- Weighted Average Costs of capital (WACC) [min, average, and max]
- Debt share (DS) [min, average, and max]
- DSCR requirements [min, average, and max]
- Loan tenors (LT) [min, average, and max]

***Definitions of cost of capital and financing condition values.*** A definition of the above listed inputs can be found in the following:

- Cost of debt: interest rates on project financed loans or costs of debt on a corporate level
- Cost of equity: equity return requirement, equivalent to a target equity Internal Rate of Return
- WACC: this refers to the post-tax WACC
- Debt share: the share of debt in the overall capital structure of a project financed project or within the overall capital structure of a company
  - We recommend that users not use the debt share values from the survey in combination with the DSCR requirements to reconstruct a project financing loan schedule. Instead, we recommend using the DSCR requirements as an input for a loan repayment schedule, which would result in a separate calculated debt share. Please note that this might differ from the debt share that has been reported by the survey.
    We recommend this approach because the DSCR requirements and debt shares were collected as estimates. While these have in many cases referred to specific projects, the interviewees did not read them from a specific project financing term sheet. Instead, they recollected them from memory. Therefore, in cash flow optimization exercises (for instance where Excel Solver is used to calculate bid levels, while using the financing variables as inputs), one arrives at debt shares that differ from the surveyed ones.
- DSCR requirement: the DSCR requirement level that banks typically define as part of their loan agreement with a client. Within a project financing capital structure, this value is used to sculpt the loan repayment schedule. Based on a projection of future Cash Flow Available for Debt Service and a probabilistic production scenario (typically p-90), the bank determines periodic interest and principal repayments
  - Within the survey, we did not ask for a corresponding production scenario, when asking the interviewees for a DSCR requirement. We decided against detailing this question further to avoid a lower response rate from interviewees. Data users should take this into account when using the data. We recommend that users re-construct the corresponding p-values using these threshold values:
    0 - 1.2: p-90
    1.2 - 1.3: p-75
    1.3 - : p-50
- Loan tenor: the length of the loan repayment period of loan maturity

The data contains combined values for both project financed and balance sheet financed projects.

For a more detailed discussion and definitions of these financing conditions and costs of capital values, please refer to the AURES II publication titled “Effect of auctions on financing conditions for renewable energy projects” ( Ðukan *et al.*, 2019).

**Figure 1.**
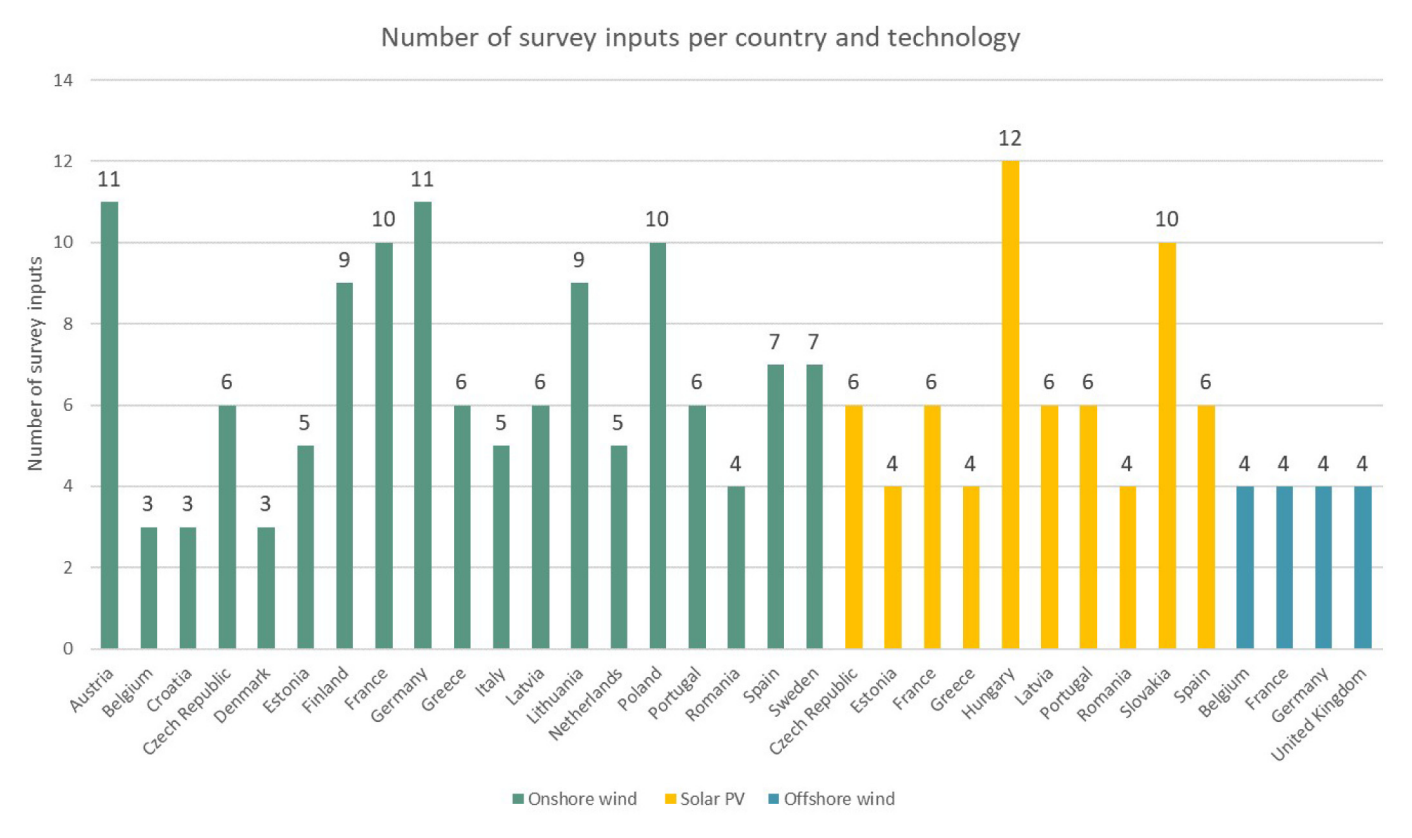
Geographical distribution and number of data inputs per country and technology.

**Figure 2.**
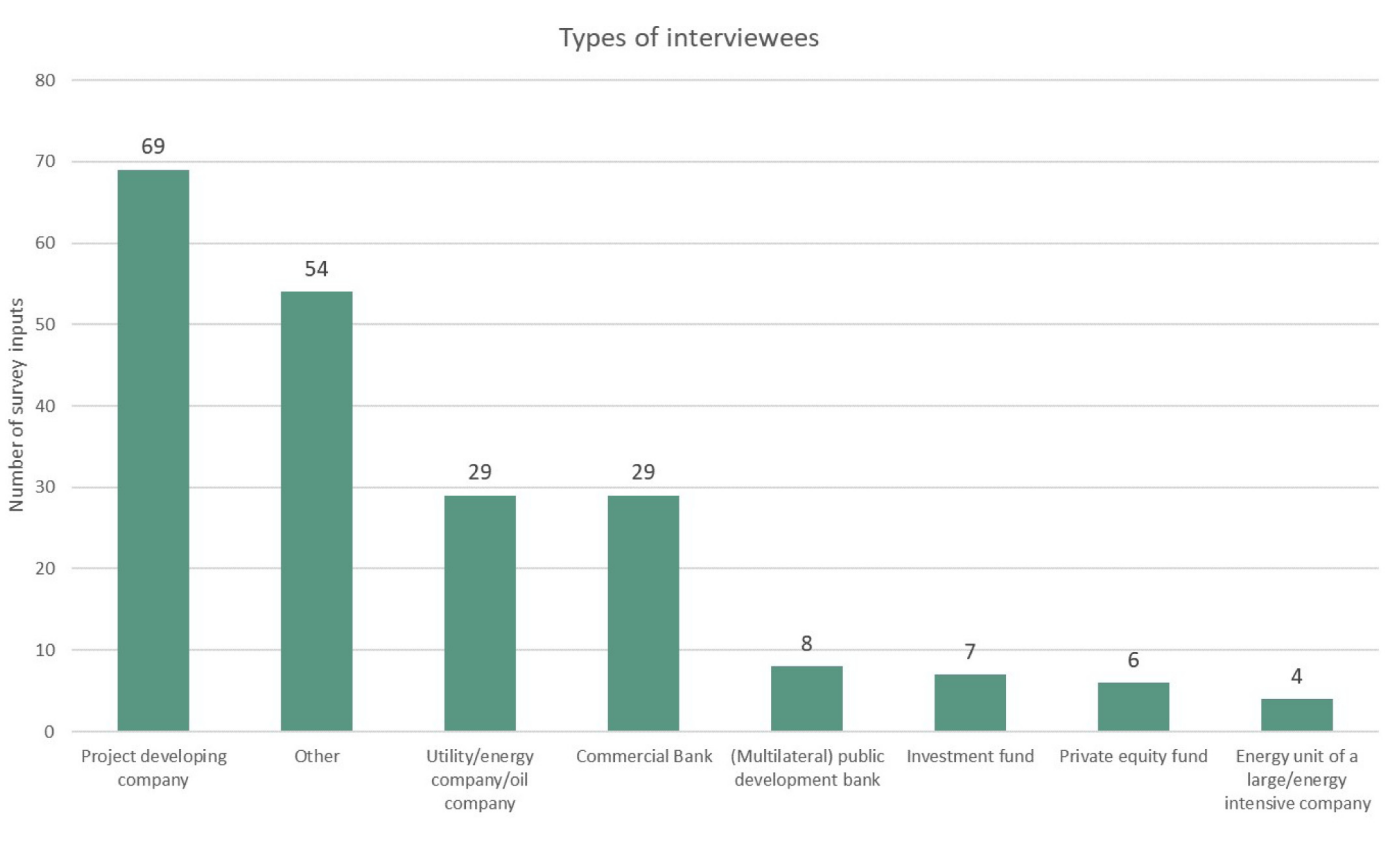
Types of interviewees by professional association.

**Figure 3.**
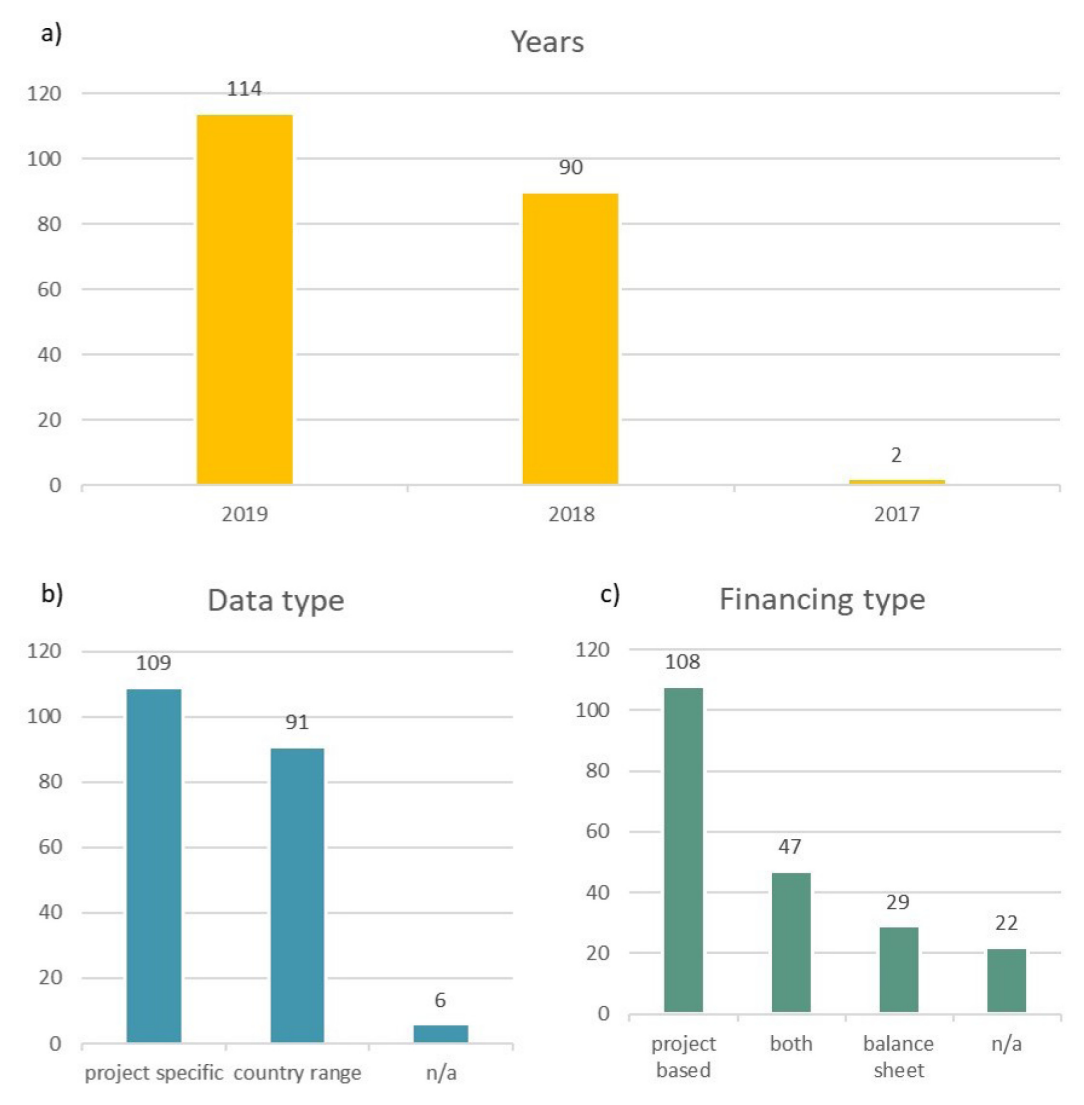
**a**) years data inputs relate to **b**) data input types **c**) financing types of data inputs.

***Survey inputs – qualitative descriptions.*** Regarding the exact geographic distribution of the surveyed countries and the number of survey inputs per country, a summary in Figure 1 is provided. Further, Figure 2 shows the types of interviewees according to their professional association. Most of the interviewees (62%) were either project developers, representatives of utilities and energy companies, or commercial bankers. Finally, Figure 3 shows in a) the years that the data inputs relate to, b) whether the data inputs relate to a specific project or if they are country estimates and c) whether the data inputs related to either project financed projects or balance sheet financed projects. Almost all (99%) of the data relate to years 2019 and 2018, and 53% of the data are project specific while the rest are mostly country estimates. Finally, 52% of the dataset relate to project financed projects.

### Dataset validation

In order to validate the data, a first overview of the results was plotted and sent out to all interviewees asking for feedback (European map with average WACC values). Some of the interviewees provided qualitative inputs via e-mail, such as confirmation of the values presented and plausible explanations behind the collected data, for instance, the role of interest rates, the competition between market players, etc. These inputs were collected and compiled into an Excel 2016 file.

Afterwards, the research team conducted two internal workshops to discuss not only the results obtained and the common challenges encountered, but also the feedback received from some of the interviewees. The results of these workshops were used as qualitative inputs to analyse and understand the data better. The results from the workshops were verbal comments from the participants of the workshops. Most of them were questions on particular data points or they were comments on the overall explanation of the historic development of the data (the decrease of the cost of capital). The comments from the workshops were not recorded in a systematic manner. Therefore, extended data does not exist. The quantitative values were not modified, but the feedback and workshops were helpful to identify trends behind the observed values and test hypothesis explaining those values.

Certain Member States have had very little or no development of wind power projects in the period 2017–2019. Hence, the data of these countries could be less representative. To assure transparency and reliability, the results and graphs included in the AURES II report ( Roth *et al.*, 2021) account for this issue. In the European maps, where the data is presented, countries that have had less than 3% wind power capacity increase between 2017–2019 have their geographical borders outlined in red. To measure the wind power capacity increase, the data from EurObservER (2020) was used. Highlighting the countries with a low increase in installed wind energy capacity indicates that the survey data inputs in these cases may refer to older market data.

## Data availability

### Underlying data

Zenodo: Financing conditions of renewable energy projects – results from an EU wide survey (1.2) ( Brückmann *et al.*, 2021)

DOI: https://doi.org/10.5281/zenodo.5572754

This file contains the following underlying data:

- Costs of capital and financing conditions for renewable energy in Europe - annotated version. (This .xlsx file includes the data, as well as comments and explanations for a better understanding of the data provided.)
- Costs of capital and financing conditions for renewable energy in Europe - clean version. (This .csv file includes only the data without any explanations and comments and has been modified to be directly used in data processing software.)

Data are available under the terms of the “Creative Commons Attribution 4.0 International license” (CC-BY 4.0).

### Extended data

Zenodo: Financing conditions of renewable energy projects – results from an EU wide survey (1.2) ( Brückmann *et al.*, 2021)

DOI: https://doi.org/10.5281/zenodo.5572754

This file contains the following extended data:

- Original WACC questionnaire for exploratory interviews. (This .pdf file contains the original version of the questionnaire which was used only for the exploratory interviews.)
- WACC questionnaire for focus countries. (This .pdf file contains the final questionnaire used for the five focus countries.)
- WACC questionnaire for non-focus countries. (This .pdf file contains the final questionnaire used for the non-focus countries.)

Data are available under the terms of the “Creative Commons Attribution 4.0 International license” (CC-BY 4.0).

## References

[ref-1] Brückmann R Roth A Jimeno M : Financing conditions of renewable energy projects – results from an eu wide survey.(1.2) [data set],2021. 10.5281/zenodo.5572754 PMC10446053 37645147

[ref-3] Ðukan M Kitzing L Brückmann R : Effect of auctions on financing conditions for renewable energy. a mapping of auction design and their effects on financing.2019. Reference Source

[ref-4] EurObservER: Wind energy barometers.2020. Reference Source

[ref-2] Roth A Brückmann R Jimeno M : Renewable energy financing conditions in Europe: survey and impact analysis. Insights on cost of capital, significance of explanatory variables, and cash-flow impacts on support cost in auction and non-auction environments.2021. Reference Source

