# Supplementary material for: Financing conditions of renewable energy projects – results from an EU wide survey
Source: Open Res Eur. 2022 Jun 20;1:136. Originally published 2021 Nov 12. [Version 2] doi: 10.12688/openreseurope.13969.2 (PMC10446053; doi:10.12688/openreseurope.13969.2)
Supplement: Peer review history

# Peer review: Reviewer response for version 2

**DOI:** 10.21956/openreseurope.16004.r29563

**Published:** 2022-06-27

**Authors:** Farid Karimi

**Affiliations:** 1 Novia University of Applied Sciences, Raseborg, Finland

The article was appropriately revised and addressed my comments substantially. Thus, I deem it acceptable. I thank the authors for addressing the comments.

Are sufficient details of methods and materials provided to allow replication by others?

Yes

Is the rationale for creating the dataset(s) clearly described?

Yes

Are the datasets clearly presented in a useable and accessible format?

Partly

Are the protocols appropriate and is the work technically sound?

Yes

Reviewer Expertise:

Social sciences of an energy transition using both qualitative and quantitative methods particularly interviews data collection and analysis.

I confirm that I have read this submission and believe that I have an appropriate level of expertise to confirm that it is of an acceptable scientific standard.

# Peer review: Reviewer response for version 1

**DOI:** 10.21956/openreseurope.15054.r29019

**Published:** 2022-05-04

**Authors:** Farid Karimi

**Affiliations:** 1 Novia University of Applied Sciences, Raseborg, Finland

Financing sustainable energy projects is a critical issue for expediting the energy transition. This work presents worthy data that could be used in several fields of research and also beyond academia. This data note is presented in a clear and transparent manner. The authors follow fairly all necessary technical and ethical rules for conducting interviews. However, the data note needs some minor revisions to my point of view:

- Page 3, *Defining the scope*, the second and the third paragraphs are a bit unclear and contradictory. In the second paragraph, it is mentioned that “only countries that had conducted at least one renewable energy auction were considered”. However, in the third paragraph, it is mentioned that “At the moment of selecting the focus countries, the following Member States had already conducted auctions…..From these countries, the research team selected the focus countries considering a geographical balance.” However, some countries in these two categories are the same (e.g., Denmark and Germany).
- Please clarify if the interviewees only expressed their organisational views or if they additionally could express their personal views as well in answering the questions.
- It is worthwhile to elaborate a bit more on the category of “other” in order to clarify who were categorised under this title. This is not clear.
- It is worthwhile to add a figure to demonstrate how many of the interviews represented private sectors.
- For data validation, the authors used workshops. However, it is not clear who participated in these “internal” workshops. By “internal, "I suspect that only the researchers involved in this project participated. If this is the case, there is a need for clarification on how these workshops, without the participation of other stakeholders or the interviewees, contribute to the data validation.

Are sufficient details of methods and materials provided to allow replication by others?

Yes

Is the rationale for creating the dataset(s) clearly described?

Yes

Are the datasets clearly presented in a useable and accessible format?

Partly

Are the protocols appropriate and is the work technically sound?

Yes

Reviewer Expertise:

Social sciences of an energy transition using both qualitative and quantitative methods particularly interviews data collection and analysis.

I confirm that I have read this submission and believe that I have an appropriate level of expertise to confirm that it is of an acceptable scientific standard, however I have significant reservations, as outlined above.

# Response

**Published:** 2022-05-16

**Authors:** Agustin Roth

**Affiliations:** eclareon GmbH, Germany

*We would like to thank the reviewer for their positive feedback on our work and are very grateful for their constructive comments and suggestions. They most certainly helped to improve the quality of our data note.  * Financing sustainable energy projects is a critical issue for expediting the energy transition. This work presents worthy data that could be used in several fields of research and also beyond academia. This data note is presented in a clear and transparent manner. The authors follow fairly all necessary technical and ethical rules for conducting interviews. However, the data note needs some minor revisions to my point of view:

- Page 3, Defining the scope, the second and the third paragraphs are a bit unclear and contradictory. In the second paragraph, it is mentioned that “only countries that had conducted at least one renewable energy auction were considered”. However, in the third paragraph, it is mentioned that “At the moment of selecting the focus countries, the following Member States had already conducted auctions…..From these countries, the research team selected the focus countries considering a geographical balance.” However, some countries in these two categories are the same (e.g., Denmark and Germany).

*Author Response: We included a more substantiate explanation of the criteria used for selecting the focus countries in the manuscript and adapted the structure of this part to make it clearer.*

- Please clarify if the interviewees only expressed their organisational views or if they additionally could express their personal views as well in answering the questions.

*Author Response: The interviewees did not represent their companies but were rather interviewed as "experts". Some of those even clearly stated that the answers were their personal opinions and not necessarily the ones of their companies. Therefore, we included the following footnote in the manuscript: "In addition, the survey inputs are the personal expert assessments of the interviewees and do not necessarily represent the official views of their organisations."*

- It is worthwhile to elaborate a bit more on the category of “other” in order to clarify who were categorised under this title. This is not clear.

*Author Response: We included a more complete definition for the category "other", as follows: "Under this category, we included among others 1) (independent) consultants and experts, 2) RES associations and chambers, and 3) RES plant operators."*

- It is worthwhile to add a figure to demonstrate how many of the interviews represented private sectors.

*Author Response: We included the following sentence in the manuscript: "Furthermore, almost all of the survey inputs (except 8 out of 206 data points) came from stakeholders from the private sector."*

- For data validation, the authors used workshops. However, it is not clear who participated in these “internal” workshops. By “internal, "I suspect that only the researchers involved in this project participated. If this is the case, there is a need for clarification on how these workshops, without the participation of other stakeholders or the interviewees, contribute to the data validation

*Author Response: One the one hand, we performed an external validation by sending our (aggregated) findings to all the interviewees for feedback. In addition, we performed an internal quality check with all the involved interviewers. We added two subheadings ("External validation" and "Internal quality check") in the manuscript to make it clearer.*

# Peer review: Reviewer response for version 1

**DOI:** 10.21956/openreseurope.15054.r28841

**Published:** 2022-04-11

**Authors:** Fang Zhang

**Affiliations:** 1 Tsinghua University, Beijing, China

The data note is a timely work for a well understanding of financial conditions in Europe. It is always challenging to gather data about financial aspects (e.g. debt or equity costs) of renewable energy projects as it could be commercially confidential. I think the research teams did a nice job to ensure the quality of data collection. And it also takes necessary steps to protect the data from its interviewees. I only have several suggestions for further consideration.

1. I do think project sizes matter a lot for the financial conditions of renewable energy projects. It would be helpful if the data note could clarify the sizes of projects that they are surveying.
2. Since “others” ranks as the second largest group of interviewees. The note should elaborate more whom are covered under others besides consultants.
3. The data collection made a division between focus and non-focus countries but the data collection covered all these countries. I would appreciate if the authors could explain the rationales behind this distinction.

Are sufficient details of methods and materials provided to allow replication by others?

Yes

Is the rationale for creating the dataset(s) clearly described?

Yes

Are the datasets clearly presented in a useable and accessible format?

Yes

Are the protocols appropriate and is the work technically sound?

Yes

Reviewer Expertise:

clean energy innovation, climate finance, and climate policy analysis

I confirm that I have read this submission and believe that I have an appropriate level of expertise to confirm that it is of an acceptable scientific standard.

# Response

**Published:** 2022-05-16

**Authors:** Agustin Roth

**Affiliations:** eclareon GmbH, Germany

*We would like to thank the reviewer for their positive feedback on our work and are very grateful for their constructive comments and suggestions. They most certainly helped to improve the quality of our data note. Please find our answers below.*

 The data note is a timely work for a well understanding of financial conditions in Europe. It is always challenging to gather data about financial aspects (e.g. debt or equity costs) of renewable energy projects as it could be commercially confidential. I think the research teams did a nice job to ensure the quality of data collection. And it also takes necessary steps to protect the data from its interviewees. I only have several suggestions for further consideration.

1. I do think project sizes matter a lot for the financial conditions of renewable energy projects. It would be helpful if the data note could clarify the sizes of projects that they are surveying.

*Author Response: We added the following sentence: "It should be noted that for the focus countries, the data relates to the following project sizes: for onshore wind (Germany 5-36 MW, Denmark 20-100 MW, Greece 2.7-83 MW, Portugal 500 MW, Spain 70-500 MW), solar PV (Greece 10-50 MW, Portugal 500 MW, Spain 500 MW), offshore wind (Germany 140-600 MW)."*

1. Since “others” ranks as the second largest group of interviewees. The note should elaborate more whom are covered under others besides consultants.

*Author Response: We included a more complete definition for the category "other", as follows: "Under this category, we included among others 1) (independent) consultants and experts, 2) RES associations and chambers, and 3) RES plant operators."*

1. The data collection made a division between focus and non-focus countries but the data collection covered all these countries. I would appreciate if the authors could explain the rationales behind this distinction

*Author Response: We included a more substantiate explanation of the criteria used for selecting the focus countries in the manuscript and adapted the structure of this part to make it clearer.*
